# Curcumin: Updated Molecular Mechanisms and Intervention Targets in Human Lung Cancer

**DOI:** 10.3390/ijms13033959

**Published:** 2012-03-22

**Authors:** Ming-Xiang Ye, Yan Li, Hong Yin, Jian Zhang

**Affiliations:** 1 Department of Pulmonary Medicine, Detection Center of Tumor Biomarker, Xijing Hospital, Fourth Military Medical University, 710032 Xi’an, China; E-Mail: mingxiangye@yahoo.cn; 2 Department of Pulmonary Medicine, First People’s Hospital of Xi’an, 710032 Xi’an, China; E-Mail: huliyan1971@126.com; 3 Department of Radiology, Medical Imaging Center, Xijing Hospital, Fourth Military Medical University, 710032 Xi’an, China

**Keywords:** lung cancer, curcumin, EGFR, miRNA, autophagy, cancer stem cell

## Abstract

Curcumin, a yellow pigment derived from *Curcuma longa Linn*, has attracted great interest in the research of cancer during the past decades. Extensive studies documented that curcumin attenuates cancer cell proliferation and promotes apoptosis *in vivo* and *in vitro*. Curcumin has been demonstrated to interact with multiple molecules and signal pathways, which makes it a potential adjuvant anti-cancer agent to chemotherapy. Previous investigations focus on the mechanisms of action for curcumin, which is shown to manipulate transcription factors and induce apoptosis in various kinds of human cancer. Apart from transcription factors and apoptosis, emerging studies shed light on latent targets of curcumin against epidermal growth factor receptor (EGFR), microRNAs (miRNA), autophagy and cancer stem cell. The present review predominantly discusses significance of EGFR, miRNA, autophagy and cancer stem cell in lung cancer therapy. Curcumin as a natural phytochemicals could communicate with these novel targets and show synergism to chemotherapy. Additionally, curcumin is well tolerated in humans. Therefore, EGFR-, miRNA-, autophagy- and cancer stem cell-based therapy in the presence of curcumin might be promising mechanisms and targets in the therapeutic strategy of lung cancer.

## 1. Introduction

Lung cancer is the leading cause of cancer related mortality worldwide, with approximately one point six million new cases and one point four million deaths each year [1]. The prognosis of lung cancer is poor because lung cancer can be symptomless in the early stage and the patients usually consult clinicians when distant metastases are already present. Over the past decades, lung cancer has switched from an untreatable disease with a dismal outcome to one that has increasing numbers of chemotherapeutic regimens and individualized treatment options available. There has been a significant improvement in the overall survival time for lung cancer patients since the emergence of well-established systemic chemotherapy strategies and newer approaches to achieve disease control. However, chemotherapy as the backbone for lung cancer has shown weak therapeutic results because of deleterious adverse effects and the possibility of cancer recurrence after drug withdrawal. In addition, the development of multidrug resistance has greatly limited the life-expanding effects of chemotherapeutic agents with less than 15% patients reaching five years of survival [2]. Therefore, searching new therapeutic agents and exploring novel intervention targets might provide more clinical benefits and indicate better outcomes in lung cancer therapy.

Having established chemopreventive activities and preclinical anti-cancer effects, numerous naturally occurring phytochemicals provide novel therapeutic approaches and treatment alternatives. Vinblastine from *Vinca rosea* is one of the earliest examples that originated from medical herbs prescribed for cancer, and paclitaxel is perhaps one of the most recent examples that originates from a Chinese pacific yew plant. Other plant-derived anti-cancer agents include etoposide, teniposide, homoharringtonine and camptothecin derivatives. These bioactive phytochemicals exert their anti-cancer activities mainly through inducing cell cycle arrest and triggering cancer cell apoptosis. Among nearly 600 kinds of phytochemicals, curcumin (diferuloylmethane), a yellow spice and phenolic compound derived from the plant *Curcuma longa*, is one of the most powerful and promising chemopreventive and anti-cancer agents. Both epidemiological evidence and clinical trials have demonstrated that the consumption of a curcumin-rich diet has is inversely correlated with certain types of human malignancies [3–5]. Extensive studies have revealed the mechanisms of action of curcumin as an anti-cancer agent, in which NF-κB, STAT3, COX-2, NOS, ROS, Akt (defined as transcription factors), anti-apoptotic proteins, growth factor receptor and multidrug-resistance proteins (defined as apoptosis related molecules) are involved [6–11]. The rapid evolution in cancer research has driven a new understanding of cancer biology and set novel therapeutic targets. Meanwhile, a growing number of evidence shows that curcumin affects many more targets and multiple mechanisms in cancer cells.

**Figure 1:**
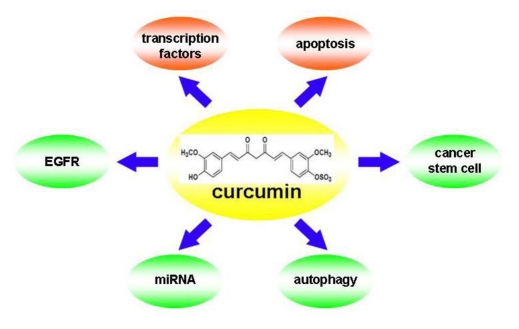
Currently well established targets (red bubbles) and potential novel targets (green bubbles) for curcumin in human lung cancer.

The present review focuses on the roles of epidermal growth factor receptor (EGFR), microRNAs (miRNAs), autophagy and cancer stem cells in lung cancer therapy, and provides a synopsis of the association of curcumin and these recently established targets (Figure 1). Therapeutic strategies being implemented to target EGFR, miRNAs, autophagy and cancer stem cells are discussed, including the use of curcumin in lung cancer.

## 2. Curcumin and Acquired Epidermal Growth Factor Receptor (EGFR)-Tyrosine Kinase (TKIs) Resistance

**Figure 2:**
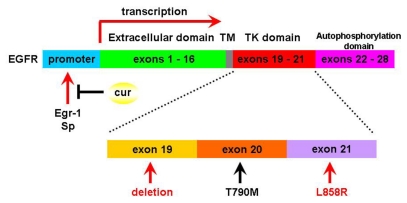
The gene structure of epidermal growth factor receptor (EGFR) and the genetic targets for curcumin and epidermal growth factor receptor-tyrosine kinase (EGFR-TKIs). Exons 1–16 encode the EGFR extracellular domain (green), exons 17–18 encode the transmembrane domain (gray) and exons 19–21 encode the tyrosine kinase domain (red). Cancer cells harboring deletions in exon 19 or L858R mutation in exon 21 are sensitive to EGFR-TKIs (red arrows indicated). Secondary T790M mutation in exon 20 is a mechanism of acquired EGFR-TKIs resistance (black arrow indicated). Two transcription factors Egr-1 and Sp bind to EGFR gene promoter and accelerate EGFR mRNA transcription. Whereas curcumin attenuates Egr-1 and Sp binding and inhibites EGFR mRNA expressions.

Epidermal growth factor receptor (EGFR) is a member of the family of growth factor receptors tyrosine kinases (TKs) that play a crucial role in cell proliferation, division and differentiation. Upon EGFR activation, the downstream intracellular signaling cascades such as the PI3K/Akt, Raf/MEK/Erk, and STAT pathways are subsequently activated leading cell survival. Aberrant EGFR expression and signaling are found in various kinds of human cancers. Somatic mutations in exon 19 and exon 21 encoding the EGFR TKs domain are found in about 10% of non-small cell lung cancer (NSCLC) in the United States, with 78.8% frequency in the Chinese populations with lung adenocarcinoma [12]. NSCLC patients harboring exon 19 deletion or exon 21 L858R somatic mutations experience significant tumor regression when prescribed the reversible small molecular tyrosine kinase inhibitors (TKIs) such as gefitinib and erlotinib. Discovery of EGFR-TKIs which is based on the novel understanding of biological mechanisms underlying lung cancer development is a breakthrough in the therapeutic strategy of thoracic oncology. The median times to progression in the advanced NSCLC range from 9.4 to 13.3 months after EGFR-TKIs treatment is three- to fourfold greater than with conventional platin-based chemotherapy [13–15]. Unfortunately, nearly all NSCLC patients who possess activating mutations and initially respond well to EGFR-TKIs, eventually procede to disease progression within 6–12 months after EGFR-TKIs treatment. This acquired EGFR-TKIs resistance is closely associated with a secondary mutation in EGFR. EGFR T790M (threonine to methionine mutation at codon 790 of EGFR) gatekeeper mutation is the most common mechanism of acquired resistance to EGFR-TKIs and it has been detected in over 50% of resistant NSCLC patients and from EGFR-TKIs resistant cell lines [16–18] (Figure 2). The precise mechanisms of EGFR T790M-mediated acquired EGFR-TKIs resistance are not yet fully understood. To date, EGFR T790M leads to steric hindrance and interferences with the EGFR-TKIs binding in the ATP kinase binding pocket [19]. In this respect, it has been shown that second generation irreversible EGFR-TKIs, including HKI-272, BIBW2992 and PF00299804, partly overcome acquired resistance in cell lines harboring EGFR T790M in several pre-clinical studies [20–22].

Another mechanism related to acquired EGFR-TKIs resistance involves amplification of MET, which has been reported in around 20% of acquired resistant patients. MET protein is a TK that is over expressed in various types of cancer cells, including lung cancer. The ability of MET to induce EGFR-TKIs resistance was correlated well with its mitogenic, motogenic, morphogenic, and anti-apoptotic properties [23,24]. Continuous administration of gefitinib or erlotinib in lung cancer patients with MET amplification did not interrupt tumor progression, regardless of EGFR status. Conversely, several MET inhibitors promoted NSCLC cell apoptosis and potentially reversed EGFR-TKIs resistance [25,26]. In a study performed by Yano *et al.*, the authors demonstrated that MET restored persistent PI3K/Akt signaling pathways, independent of EGFR status, thus inducing gefitinib resistance of lung cancer cells bearing EGFR-activating mutations [27]. In this regard, MET inhibitors have raised a broader interest in their therapeutic indications in cancer. In particular, Engelman *et al.* showed that a MET inhibitor PHA-665752 could restore cellular sensitivity to EGFR-TKIs [28].

Both established resistant mechanisms lead to sustained TKs downstream PI3K/Akt signaling in the presence of EGFR-TKIs [29,30]. Therefore, concomitant inhibition of EGFR and MET is required to eliminate the resistant cells and reverse EGFR-TKIs resistance. Treatment of cancer cells with curcumin results in a dose- and time-dependent inhibition of cell proliferation associated with curcumin’s epigenetic modulation of target genes and proteins [31–33]. Existing evidence suggest that curcumin acts as an epigenetic agent through interaction with histone deacetylases, histone acetyltransferases and DNA methyltransferases [34]. However, the precise mechanisms by which curcumin modulates gene expression have not yet been fully delineated. One potential theory is that curcumin produces integrated modulation of intracellular signal transduction and transcription factors. Nuclear factor kappaB (NF-κB) is a generally accepted predominant epigenetic target of curcumin. Numerous lines of evidence have suggested curcumin suppressed cancer cell proliferation by reducing NF-κB signaling and its genetic targets, including cyclin D1, c-myc, Bcl-2, Bcl-xl, CIAP-1, COX-2, VEGF and matrix metalloproteinase (MMP) [35,36]. Activated protein-1 (AP-1) is a transcription factor that transactivates c-met promoter. C-met gene expression is also inducible by its ligand hepatocyte growth factor (HGF). Seol *et al.* reported that curcumin inhibited AP-1 activity and blocked its transactivation of the c-met promoter. Moreover, induction of the endogenous c-met gene by HGF was inhibited by the addition of curcumin as well [37]. Down-regulation of c-met by administration of curcumin might provide potential targets to overcome MET-dependent EGFR-TKIs resistant mechanisms.

For EGFR mRNA and protein, several other transcription factors have been recently recognized. Curcumin dose-dependently suppressed EGFR mRNA and protein expressions in three lines of human colon cancer-derived cells. Luciferase reporter assay and electrophoretic mobility shift assay indicated that curcumin interrupted trans-activation activity of transcription factor Egr-1 in the EGFR promoter as a putative mechanism in regulating EGFR expression [38]. Similarly, curcumin repressed transcription factor Sp, which promoted EGFR expression, and showed a synergistic effect with gefitinib in KU7 and 253JB-V human bladder cancer cells [39]. Collectively, these reports highlighted the epigenetic profile of curcumin and supported it as a potent modulator for EGFR transcription factors. Suppression of EGFR mRNA and its protein product using curcumin’s epigenetic activity would block the downstream tyrosine kinase signaling cascade and overcome acquired EGFR-TKIs resistance (Figure 2). Compared with inhibiting EGFR downstream signal transduction by EGFR-TKIs, direct removal of this receptor seems to offer more therapeutic effects. And this newly established anti-cancer mechanism of curcumin might provide more clinical benefits especially in lung cancer, regardless of *EGFR* mutation status.

Conversely, in a recent study, Lee and colleagues investigated the EGFR degradation-inducing potential of curcumin in the primary and acquired EGFR-TKIs resistant cell lines (CL1-5 and A549, EGFR wide type; H1975, EGFRL858R + T790M). The IC50 of gefitinib for these resistant cells at 72 h (15–20 μM) was up to nearly 700-fold when compared with the EGFR-TKIs sensitive PC-9 cells (EGFR19del) (IC_50_ = 30 nM). The resistant cell proliferation was significantly inhibited when gefitinib and curcumin were used in combination. Pre-treatment with curcumin also dose-dependently reduced phosphor-EGFR protein expression, and its downstream signaling was stimulated by exogenous EGF. Interestingly, the endogenous EGFR was dramatically depleted in response to curcumin in the three gefitinib resistant cells as well [40]. Immuno-precipitation data showed that curcumin induced EGFR protein degradation by accelerating ubiquitin-proteasome ability in wide type EGFR (CL1-5 and A549) and also in the mutated EGFR with T790M (H1975). In further support of the data, when experiments were performed in tumor-bearing SCID mice, administration of curcumin inhibited growth of CL1-5, A549 and H1975 xenografts and enhanced the anti-tumor efficiency of gefitinib. Curcumin simultaneously down-regulated oncogenic protein expression, in particular Akt and c-MET in CL1-5 xenograft models. Since EGFR-TKIs inevitably caused undesirable gastrointestinal side effects, gefitinib single agent in combination with curcumin therapies were evaluated for potent intestinal damage *in vivo* and *in vitro*. Combined treatment reduced gefitinib-induced villi damage and apoptosis in mice intestines [40].

**Figure 3:**
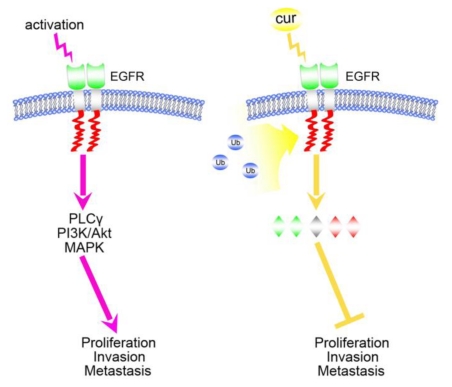
Molecular mechanisms of EGFR-mediated cancer cell proliferation, invasion and metastasis. Upon activation, the extracellular domain of EGFR protein (green) transducts the signal into the intracellular tyrosine domain (red) through the transmembrane domain (gray). The PLCγ, PI3K/Akt and MAPK pathways are subsequently activated, leading to cancer cell proliferation, invasion and metastasis. However, in the presence of curcumin, EGFR protein undergoes ubiquitination and degradation. Decreased EGFR protein on the cell membrane attenuates proliferative, invasive and metastatic signals, leading to cancer cell apoptosis and death. This mechanism works regardless of EGFR mutation status.

Taken together, all of the abovementioned studies indicate a potent role of curcumin in overcoming resistance supported by EGFR-TKIs in NSCLC. Herein we raised the hypothesis that curcumin as an adjuvant therapeutic agent in both primary and secondary EGFR-TKIs resistant NSCLC cells, at least in part, for its epigenetic activity on EGFR expression. Promoting ubiquitin-proteasome-mediated EGFR degradation and inhibiting EGFR downstream signaling initiated by curcumin are yet novel strategies that ultimately might be embraced as potential therapies for resistant NSCLC (Figure 3). Co-administration of curcumin and EGFR-TKIs attenuated the adverse gastrointestinal effects of EGFR-TKIs as well as its ability to function as a chemosensitizer in NSCLC.

## 3. Curcumin and Its Epigenetic Actions on MicroRNAs

MicroRNAs (miRNAs) are a class of short highly conserved non-coding RNAs, which undergo sequential processing by the RNase III-like enzymes Drosha/Dicer from hairpin-structured precursors [41]. Mature miRNAs regulate multiple gene expression by imperfect complementary binding to target mRNAs at the 3′-untranslated region (UTR), leading to mRNA cleavage or translational repression [42]. Several computational algorithms have been developed to predict transcript targets of miRNAs. *In silico* analysis suggests that miRNAs may be responsible for the regulation of up to one third of the genome simultaneously [43]. To date, over 800 annotated miRNAs have been identified in humans, yet the exact biologic relevance and functional targets of a wide range miRNAs remain undefined. Numerous evidence has revealed miRNAs emergence as key posttranscriptional regulators of gene expression and regulation of a variety of biological processes, including development, cell proliferation, differentiation and apoptosis [44,45]. First experimental evidence that suggested that miRNAs might participate in human carcinogenesis and hit specific molecular targets came from the realization of the chromosomal deletion (13q14.3) in human chronic lymphocytic leukemia (CLL), in which two miRNAs, miR-15a and miR16-1, were down-regulated in 68% CLL cases [46]. Following this, a growing number of findings have also shown that miRNAs are implicated in all stages of cancer, from initiation to promotion and progression, and act as tumor suppressors or oncogenes [47–49]. Certain miRNAs are up-regulated or down-regulated in human cancer, with some overlapping miRNA profiles depending on tissue origin. For example, miRNA *let-7*, targeting the oncogene *ras*, was down-regulated [50], while the miR-17-92 cluster at chromosome 13q31.3 was reported to be over-expressed in lung cancer [51,52].

Aberrant miRNAs signatures have been observed in various kinds of human cancers. More recently, miRNAs have been detected in sputum and peripheral blood, which gave rise to a lot of attention for their potential clinical relevance in the early detection and prognostic prediction of lung cancer [53–56]. Forced expression or competitive suppression of aberrant miRNA can regulate the biological alteration during carcinogenesis, underscoring the therapeutic potential of miRNAs in lung cancer. Takamizawa *et al.* [50] firstly reported reduced expression of *let-7* in human NSCLC specimens as well as in cell lines, and loss of *let-7* was recognized as a marker for poor prognosis. Renewed or ectopic expression of *let-7* in lung cancer cell lines inhibited cell growth. Conversely, the cluster miR-17-92 was up-regulated in lung cancer cells with neuroendocrine properties, in particular in small cell lung cancer [51]. Over-expression of miR-17-92 promoted cancer cell proliferation via interaction with tumor suppressors and transcription factors. Repression of miR-17-92 suppressed its oncogenic effects and elicited lung cancer cell death in cell lines [57]. These data indicate that therapeuticalyy inhibiting or restoring aberrantly under-expressed miRNAs in miRNAs-based strategies might provide potential clinical benefits as a new generation of therapeutics.

Only a few reports have so far investigated the effect of curcumin on miRNAs expressions. A pioneering study from Sun *et al.* [58] identified for the first time that curcumin could modulate miRNAs signatures in a human pancreatic carcinoma BxPC-3 cell line. They found that miR-22 was the most up-regulated and miR-199a* the most suppressed. The major challenge for miRNAs studies is to identify the biologically relevant downstream targets that they regulate. The expression of two computationally predicted targets for miR-22, SP1 transcription factor (SP1) and estrogen receptor 1 (ESR1), were examined. Forced expression of miR-22 by either treatment with curcumin or miR-22 mimetics transfection inhibited the expression of SP1 and ESR1 target genes, whereas miR-22 anti-sense enhanced SP1 and ESR1 expressions [58]. Shortly after Sun and colleagues’ report, we identified miRNAs signature profiles as a response to curcumin in human lung cancer by high-throughput microarray [59]. After 48 h of curcumin treatment at the concentration of 15 μmol/L, we observed that curcumin promoted A549 cell apoptosis through modulation of miR-186* and targeted its down-stream caspase-10 pathway [59]. In human colorectal cancer Rko and HCT116 cells, curcumin inhibited the transcriptional regulation of miR-21 via AP-1, suppressed cancer cell proliferation, invasion and metastasis, and stabilized the expression of the tumor suppressor programmed cell death protein 4 (Pdcd4) [60]. Likewise in breast cancer MCF-7 cells, curcumin reduced the expression of Bcl-2 and induced apoptosis by upregulating the expression of miR-15a and miR-16 [61].

Furthermore, miRNAs have been reported to modulate chemotherapeutic drug resistance. Certain miRNAs were identified that defect in apoptosis and enable cancer cells to escape apoptosis is of extremely importance. Guo *et al.* [62] found that transfection of the drug-resistant small cell lung cancer cells with the mimics of miR-134 greatly increased the sensitivity to the anti-cancer drugs cisplatin, etoposide, and doxorubicin. In another study, transfecting NSCLC with anti-miR-221 and anti-miR-222 efficiency sensitized to TRAIL, by modulating p27^kip1^ and Kit [63]. Zhu *et al.* [64] also demonstrated that enforced miR-181b expression targeting the 3'UTR of BCL-2 sensitized multidrug resistant lung cancer cells to cisplatin-induced apoptosis. These findings were extended to determine whether curcumin altered aberrantly expressed miRNAs as a chemosensitizer during acquired drug resistance. We therefore analyzed genome-wide miRNA profiling in A549/DDP multidrug-resistant human lung adenocarcinoma cells. We observed altered expressions of 342 miRNAs as a response to curcumin in A549/DDP cells, and miR-186* also emerged as the key target to reverse resistance and promote apoptosis [65]. Notably, a curcumin analogue CDF up-regulated miR-200 expression and down-regulated the expression of miR-21 in gemcitabine-resistant pancreatic cancer cells, triggering resistant cell apoptosis and down-regulation of PTEN, Akt, COX-2, prostaglandin E2, vascular endothelial growth factor, and NF-κB DNA binding activity [66] (Table 1).

Overall, the above studies support multiple roles for individual miRNAs in regulating carcinogenesis and tumor progression in various human cancers. We predict that modulation of miRNAs and their multiple target genes by curcumin is a novel therapeutic approach to human cancer with respect to promote cancer cell apoptosis and overcome drugs resistance. However, these approaches are still in the *in vitro* and preclinical stages, although intriguing evidence is emerging to support latent curcumin-miRNAs crosstalk in clinical applications. Further *in vivo* and clinical trials are essential for the confirmation of curcumin-miRNAs-based therapeutics.

## 4. Curcumin Induces Autophagy: A Double-Edged Sword in Cancer Therapy

Apoptosis has been considered as the predominant type of programmed cell death (PCD), and the major form of cell death induced by chemotherapy and radiotherapy. While autophagy has attracted a lot of interest in the field of oncological research during the past decades because it is designated as PCD type II, apoptosis is well-known as PCD type I and necrosis as type III PCD. Autophagy is an evolutionarily conserved catabolic process typically found in eukaryocytes. It is morphologically characterized by sequestering organelles and long-lived proteins in double membrane-bound autophagic vacuoles, also known as autophagosomes. These autophagosomes degrade and recycle proteins and defective cellular organelles by fusing with lysosomes to form autolysosomes [67]. Autophagy allows cells to survive in conditions of starvation by inducing lysosomal recycling of intracellular nutrients as well as caspase-independent cell death [68].

The distinct roles of autophagy in carcinogenesis as well as in cancer progression are still being elucidated, with seemingly conflicting studies suggesting that the induction of autophagy mediates chemotherapeutic and radiotherapeutic resistance in certain types of tumors while it enhances cell death in others. On the one side, decreased autophagic activity associated with malignant cells may be related to the prevention of excessive protein loss upon starvation of tumor cells [69–71]. On the other side, prolonged autophagy in cancer cells can also lead to autophagic PCD, provoking the possibility of utilizing autophagy activation for cancer therapy [72–75]. Recently, a series of published literature shed light on the study of autophagy in human lung cancer. Several natural and synthetic agents have been shown to modulate autophagy in lung cancer *in vivo* and *in vitro* [76–80]. Han *et al.* [79] indicated that lung cancer cells undergoing autophagy were resistant to EGFR-TKIs and enhanced autophagy was proposed to play a role in poor performance of EGFR-TKIs. In another study, however, autophagy facilitated cell death in lung adenocarcinoma A549 cells through interaction with p53 [81]. MG-2477 is a new anti-tubulin agent, causing A549 cells to arrest in the G2/M phase with a concomitant accumulation of cyclin B through its action on both apoptosis and autophagy [80]. Therefore, this topic has raised the question of whether manipulation of autophagy, either positively or negatively, could conceivably be applied for therapeutic strategies in lung cancer.

While autophagy appears to play dual roles in certain types of cancers as discussed above, there is also data supporting the hypothesis that the induction of autophagy can be medicated by curcumin. In human chronic myeloid leukemia (CML) K562 cell line, curcumin triggers apoptosis and autophagy in a caspase-dependent and -independent manner, respectively [81]. Alternatively, curcumin yields autophagic and non-apoptotic cancer cell death in oesophageal cancer KYSE450 and OE19 cell lines [82]. It has also been found that curcumin used as a single agent or in combination with other cytotoxic therapeutics results in accelerated autophagosome membrane protein LC3-II expression, and development of PCD in malignant glioma and hepatocellular carcinoma cell lines [83–87]. These effects are mediated through curcumin’s inhibition of the Akt/mTOR/p70S6 kinase pathway and inhibition of the ERK1/2 signaling, which are both involved in the regulation of autophagy. Activation of the Akt/mTOR/p70S6 pathway decreases curcumin-induced autophagic cell death as well as activates the ERK1/2 pathway, which results in inhibition of autophagy and induction of apoptosis [85,86,88].

Collectively, the general conclusion has been drawn from these studies that autophagy plays a dual role in determining cell survival and programmed death during its dysregulation. Results from these studies highlight the importance of curcumin in regulating autophagy and the potential utility of autophagy as a means of predicting therapeutic outcome. Although the role of curcumin-induced autophagy discussed above have not been fully investigated in lung cancers, the above-mentioned studies indicate that curcumin-induced autophagy is associated with cancer cell growth suppression and death, suggesting that its involvement in lung cancer is warranted and worth investigating.

## 5. Targeting Cancer Stem Cell

The concept of cancer stem cell (CSC) was initially introduced when Bonnet and Dick [89] published a seminal report showing that a hierarchical manner exists among leukemic cells which has gathered considerable attention over the past several years. Based on their findings, it is argued against the conventional perspectives that all cells within a cancer have equal potential to propagate the malignancy. The present understanding of CSC is that there is a limited subpopulation of primitive undifferentiated cancer cells that has the ability to self-renew, is tumorigen and invasive, undergoes asymmetrical divisions and generates all aspects of cancers. Dominant CSCs are in a quiescent state and refractory to conventional chemotherapy and radiotherapy that target dividing cells, whereas proliferative CSCs maintain and promote the development of the cancers [90–93]. CSCs have been discovered in various kinds of human cancers. Focusing on lung cancer, isolation and purification of CSCs have been identified in lung cancer cell lines as well as from patient primary samples. Enriched lung CSCs show highly metastatic and invasive properties and are very resistant to *cis*-platin, doxorubicin and ectoposide [94,95]. Among entire lung cancer cells, ALDH (aldehyde dehydrogenase)-, CD133-, OCT4- and GLDC (glycine decarboxylase)-positive cells display stem cell-like activities and interfere with these targets and have been implicated to promote CSCs differentiation as well as elimination [96–100]. Expression of these CSCs markers is linked to shorter progression-free survival in clinical protocols and thus CSCs-based therapeutics might be relevant in sensitizing the response to chemotherapy regimens and preventing cancer metastasis and relapse [101].

Given these considerations, attempts targeting CSCs and corresponding signaling pathways are being carried out in several preclinical studies. For example, RNA interference with Wnt/β-catenin signaling in lung CSCs strongly decreased cancer cell proliferation, clone formation and drug resistance abilities [100]. CSCs within cancer cell populations are resistant to chemotherapeutic agents, but salinomycin selectively kills CSCs [102]. The PTEN pathway has also been effectively targeted by specific small molecules. It has been shown that the expression of PTEN is required for stem cell maintenance while the loss of PTEN is associated with an expansion of putative CSCs population. One study on leukemia showed that PTEN knockdown lead to over-proliferation of normal hematopoietic stem cells (HSCs) and the development of myeloproliferative malignancy. The inhibitor of mTOR, sirolimus, acts on PTEN and improves healthy HSCs replication while at the same time promotes the depletion of leukemia initiating cells [103]. Of equal importance, Hedgehog, JAK-STAT, Bim, Notch and PI3K/Akt signaling also offer latent intervention targets against CSCs [104]. Curcumin as a single agent or in combination with the conventional chemotherapeutic regimen could be an effective therapeutic strategy to prevent emergence of chemoresistant cancer cells and relapse of various cancers by eliminating CSCs. Recent studies in breast cancer cells demonstrated that curcumin inhibited ALDH-expressing breast CSCs self renewal but did not cause toxicity to differentiated cells by suppressing Wnt signaling [105]. Likewise, curcumin has been shown to inhibit CD133 positive medulloblastoma, glioblastoma, pancreatic and colon CSCs proliferation through insulin-like growth factor (IGF)-, STAT3-, Hedgehog- and histone methyltransferase EZH2-dependent mechanisms [106–109].

Eliminating CSCs by targeting relevant signaling pathways and designing agents to inhibit these pathways have been somewhat successful approaches. However, these established targets participate in numerous physiological and pathological process and they are not cancers- and CSCs-specific. Systemic inhibition of these signaling pathways could have serious consequences for the host. Potential undesirable side effects caused by agents targeting the above-mentioned pathways should be kept in mind. Targeting CSCs-specific self-renewal pathways and mechanisms to delete CSCs while limiting mistargeting on normal stem cells and somatic cells might generate profound approaches to eliminate tumorigenic cells. Phytochemicals have been reported to selectively target CSCs and have no defined deleterious effect on the normal stem cells in a number of preclinical studies [110,111]. These inspiring findings highlight a research evolution of phytomedicine in the field of oncology. Further CSCs-based target therapy of phytochemicals, especially for curcumin, should be cautiously evaluated in animal models before we can suggest it for clinical trials. Optimal strategies involving combined conventional and CSCs-based therapies might kill the bulk tumor cells while simultaneously eradicating CSCs.

## 6. Perspectives and Directions

An inspiring feature of curcumin is that it is remarkable well absorbed and tolerated in animal models and humans. A growing number of systemic studies has shown that curcumin given at a dosage up to 3500 mg/kg/body weight for up to 90 days is safe in rats, dogs, or monkeys, without evident adverse effects. Curcumin has also been granted an acceptable daily intake level up to 3 mg/kg/body weight in humans by the Joint FAO/WHO Expert Committee on Food Additives [112]. Though there are few preclinical studies indicating potential liver injury, nausea and diarrhea as deleterious responses to large doses and prolonged curcumin administration, several large scale clinical trials still show that curcumin is well tolerated. A recent phase I trial in patients with advanced colorectal cancer suggested that curcumin was well tolerated at all dose levels up to 3600 mg daily for up to four months [113]. In another study by Cheng *et al.* [110] administered 500, 1000, 2000, 4000, and 8000 mg curcumin daily for three months to patients with pre-invasive malignancies and found no noticeable adverse effects. Unfortunately, curcumin undergoes a rapid metabolism and systemic elimination, and a large proportion of curcumin and its metabolites is excreted through the feces and bile, leading to a relatively poor bioavailability of curcumin *in vivo*. Curcumin orally administered at a dose of 1000 mg/kg body weight to rats resulted in 75% secretion in the feces with negligible amounts in the urine [114]. As for human studies, 2000 mg purified curcumin powder was administered to fasting volunteers and low curcumin concentrations (<10 ng/mL) in plasma were observed 1 h after administration [115]. Interestingly, the administration of curcumin in combination with 20 mg piperine, an inhibitor of glucuronidation of curcumin, significantly increased the bioavailability 20-fold [116]. In order to enhance the pharmacological activity and bioavailability, several curcumin-based chemical modifications have been explored. Formulations like curcumin nanoparticles, liposomes, micelles, phoshoplipid complexes and analogues have been demonstrated to provide longer circulation, increase the cellular permeability and induce resistance to metabolic processes [117–120].

In summary, we have discussed a line of novel molecular mechanisms and therapeutic targets for curcumin, including EGFR degradation, miRNA, autophagy and CSC, in human lung cancer. Curcumin used as a single agent or in combination with other chemotherapeutics might offer more clinical benefits through promoting EGFR degradation, manipulating miRNA expression profiles, triggering cancer cell autophagy and eliminating CSC, respectively. Since EGFR-TKIs have several advantages compared with other treatment options and they are becoming a central component of first-, second- and third-line treatment for NSCLC, reversing and overcoming EGFR-TKIs resistance are of great urgency. Given that curcumin suppresses EGFR mRNA expression, promotes EGFR protein degradation, and precludes EGFR-TKIs toxicity of intestines injury, curcumin might offer another way to attenuate EGFR-related signals regardless of EGFR mutation status. Besides, curcumin, as a novel epigenetic agent, interacts with miRNAs implicated in cell proliferation and apoptosis. Limited data are available underscoring the hypothesis that curcumin facilitates the design of appropriate therapies targeting miRNAs; more well controlled experiments are still needed to comprehensively evaluate the impact of curcumin on lung cancer outcomes in an adjuvant settings. Curcumin functional-induced cancer cell autophagy and CSC elimination are of special interest, representing an important variable to kill cancer cells and prevent cancer relapse, thereby shedding light on a new approach to the therapeutic strategy of lung cancer.

Overall, results from these studies highlight the importance of curcumin in lung cancer treatment and the potential utility of curcumin as a means of improving therapeutic outcome. Despite the potential for rationally designed therapies, confirmatory findings are required from prospective trials conducted in defined lung cancer patient populations.

## Figures and Tables

**Table 1:** Curcumin alters miRNAs and relevant target expression in pancreatic, colorectal, breast and lung cancers.

Cancer Origin	Upregulate	Downregulate	Targets	Reference
	
Pancrea	miR-22	miR-21	SP1, ESR1	[58]
miR-200		miR-199a*	PTEN	[66]
Colorectum		miR-21	AP1, Pdcd4	[60]
Breast	miR-15a		Bcl-2	[61]
	miR-16			
Lung		miR-186*	Casp-10	[59,65]

## References

[1] Ramalingam S.S., Owonikoko T.K., Khuri F.R. (2011). Lung cancer: New biological insights and recent therapeutic advances. CA Cancer J. Clin.

[2] Jemal A., Siegel R., Ward E., Hao Y., Xu J., Thun M.J. (2009). Cancer statistics, 2009. CA Cancer J. Clin.

[3] Cheng A.L., Hsu C.H., Lin J.K., Hsu M.M., Ho Y.F., Shen T.S., Ko J.Y., Lin J.T., Lin B.R., Ming-Shiang W. (2001). Phase I clinical trial of curcumin, a chemopreventive agent, in patients with high-risk or pre-malignant lesions. Anticancer Res.

[4] Dhillon N., Aggarwal B.B., Newman R.A., Wolff R.A., Kunnumakkara A.B., Abbruzzese J.L., Ng C.S., Badmaev V., Kurzrock R. (2008). Phase II trial of curcumin in patients with advanced pancreatic cancer. Clin. Cancer Res.

[5] Cruz-Correa M., Shoskes D.A., Sanchez P., Zhao R., Hylind L.M., Wexner S.D., Giardiello F.M. (2006). Combination treatment with curcumin and quercetin of adenomas in familial adenomatous polyposis. Clin. Gastroenterol. Hepatol.

[6] Chen J., Tang X.Q., Zhi J.L., Cui Y., Yu H.M., Tang E.H., Sun S.N., Feng J.Q., Chen P.X. (2006). Curcumin protects PC12 cells against 1-methyl-4-phenylpyridinium ion-induced apoptosis by bcl-2-mitochondria-ROS-iNOS pathway. Apoptosis.

[7] Divya C.S., Pillai M.R. (2006). Antitumor action of curcumin in human papillomavirus associated cells involves downregulation of viral oncogenes, prevention of NFκB and AP-1 translocation, and modulation of apoptosis. Mol. Carcinog.

[8] Chanvorachote P., Pongrakhananon V., Wannachaiyasit S., Luanpitpong S., Rojanasakul Y., Nimmannit U. (2009). Curcumin sensitizes lung cancer cells to cisplatin-induced apoptosis through superoxide anion-mediated Bcl-2 degradation. Cancer Invest.

[9] Bava S.V., Puliappadamba V.T., Deepti A., Nair A., Karunagaran D., Anto R.J. (2005). Sensitization of taxol-induced apoptosis by curcumin involves down-regulation of nuclear factor-kappaB and the serine/threonine kinase Akt and is independent of tubulin polymerization. J. Biol. Chem.

[10] Yang C.L., Ma Y.G., Xue Y.X., Liu Y.Y., Xie H., Qiu G.R. (2012). Curcumin induces small cell lung cancer NCI-H446 cell apoptosis via the reactive oxygen species-mediated mitochondrial pathway and not the cell death receptor pathway. DNA Cell Biol.

[11] Wu S.H., Hang L.W., Yang J.S., Chen H.Y., Lin H.Y., Chiang J.H., Lu C.C., Yang J.L., Lai T.Y., Ko Y.C. (2010). Curcumin induces apoptosis in human non-small cell lung cancer NCI-H460 cells through ER stress and caspase cascade- and mitochondria-dependent pathways. Anticancer Res.

[12] Sun Y., Ren Y., Fang Z., Li C., Fang R., Gao B., Han X., Tian W., Pao W., Chen H., Ji H. (2010). Lung adenocarcinoma from East Asian never-smokers is a disease largely defined by targetable oncogenic mutant kinases. J. Clin. Oncol.

[13] Rosell R., Moran T., Queralt C., Porta R., Cardenal F., Camps C., Majem M., Lopez-Vivanco G., Isla D., Provencio M. (2009). Screening for epidermal growth factor receptor mutations in lung cancer. N. Engl. J. Med.

[14] Sequist L.V., Martins R.G., Spigel D., Grunberg S.M., Spira A., Jänne P.A., Joshi V.A., McCollum D., Evans T.L., Muzikansky A. (2008). First-line gefitinib in patients with advanced non-small-cell lung cancer harboring somatic EGFR mutations. J. Clin. Oncol.

[15] Schiller J.H., Harrington D., Belani C.P., Langer C., Sandler A., Krook J., Zhu J., Johnson D.H., Eastern Cooperative Oncology Group (2002). Comparison of four chemotherapy regimens for advanced non-small-cell lung cancer. N. Engl. J. Med..

[16] Pao W., Miller V.A., Politi K.A., Riely G.J., Somwar R., Zakowski M.F., Kris M.G., Varmus H (2005). Acquired resistance of lung adenocarcinomas to gefitinib or erlotinib is associated with a second mutation in the EGFR kinase domain. PLoS Med.

[17] Kosaka T., Yatabe Y., Endoh H., Yoshida K., Hida T., Tsuboi M., Tada H., Kuwano H., Mitsudomi T. (2006). Analysis of epidermal growth factor receptor gene mutation in patients with nonsmall cell lung cancer and acquired resistance to gefitinib. Clin. Cancer Res.

[18] Uramoto H., Sugio K., Oyama T., Sugaya M., Hanagiri T., Yasumoto K. (2006). A resistance to gefitinib. Int. J. Clin. Oncol.

[19] Kobayashi S., Boggon T.J., Dayaram T., Jänne P.A., Kocher O., Meyerson M., Johnson B.E., Eck M.J., Tenen D.G., Halmos B. (2005). EGFR mutation and resistance of non-small-cell lung cancer to gefitinib. N. Engl. J. Med.

[20] Engelman J.A., Zejnullahu K., Gale C.M., Gonzales A.J., Shimamura T., Zhao F., Vincent P.W., Naumov G.N., Bradner J.E., Althaus I.W. (2007). PF00299804, an irreversible pan-ERBB inhibitor, is effective in lung cancer models with EGFR and ERBB2 mutations that are resistant to gefitinib. Cancer Res.

[21] Kwak E.L., Sordella R., Bell D.W., Godin-Heymann N., Okimoto R.A., Brannigan B.W., Harris P.L., Driscoll D.R., Fidias P., Lynch T.J. (2005). Irreversible inhibitors of the EGF receptor may circumvent acquired resistance to gefitinib. Proc. Natl. Acad. Sci. USA.

[22] Li D., Ambrogio L., Shimamura T., Kubo S., Takahashi M., Chirieac L.R., Padera R.F., Shapiro G.I., Baum A., Himmelsbach F. (2008). BIBW2992, an irreversible EGFR/HER2 inhibitor highly effective in preclinical lung cancer models. Oncogene.

[23] Birchmeier C., Birchmeier W., Gherardi E., Vande Woude G.F. (2003). Met, metastasis, motility and more. Nat. Rev. Mol. Cell Biol.

[24] Matsumoto K., Nakamura T. (2006). Hepatocyte growth factor and the Met system as a mediator of tumor-stromal interactions. Int. J. Cancer.

[25] Matsubara D., Ishikawa S., Oguni S., Aburatani H., Fukayama M., Niki T. (2010). Molecular predictors of sensitivity to the MET inhibitor PHA665752 in lung carcinoma cells. J. Thorac. Oncol.

[26] Bean J., Brennan C., Shih J.Y., Riely G., Viale A., Wang L., Chitale D., Motoi N., Szoke J., Broderick S. (2007). MET amplification occurs with or without T790M mutations in EGFR mutant lung tumors with acquired resistance to gefitinib or erlotinib. Proc. Natl. Acad. Sci. USA.

[27] Yano S., Wang W., Li Q., Matsumoto K., Sakurama H., Nakamura T., Ogino H., Kakiuchi S., Hanibuchi M., Nishioka Y. (2008). Hepatocyte growth factor induces gefitinib resistance of lung adenocarcinoma with epidermal growth factor receptor-activating mutations. Cancer Res.

[28] Engelman J.A., Zejnullahu K., Mitsuddomi T., Song Y., Hyland C., Park J.O., Lindeman N., Gale C.M., Zhao X., Christensen J. (2007). MET amplification leads to gefitinib resistance in lung cancer by activating ERBB3 signaling. Science.

[29] Engelman J.A., Jänne P.A. (2008). Mechanisms of acquired resistance to epidermal growth factor receptor tyrosine kinase inhibitors in non-small cell lung cancer. Clin. Cancer Res.

[30] Turke A.B., Zejnullahu K., Wu Y.L., Song Y., Dias-Santagata D., Lifshits E., Toschi L., Rogers A., Mok T., Sequist L.V. (2010). Preexistence and clonal selection of MET amplification in EGFR mutant NSCLC. Cancer Cell.

[31] Shishodia S., Potdar P., Gairola C.G., Aggarwal B.B. (2003). Curcumin (diferuloylmethane) down-regulates cigarette smoke-induced NF-kappaB activation through inhibition of IkappaBalpha kinase in human lung epithelial cells: Correlation with suppression of COX-2, MMP-9 and cyclin D1. Carcinogenesis.

[32] Lin S.S., Laai K.C., Hsu S.C., Yang J.S., Kuo C.L., Lin J.P., Ma Y.S., Wu C.C., Chung J.G. (2009). Curcumin inhibits the migration and invasion of human A549 lung cancer cells through the inhibition of matrix metalloproteinase-2 and −9 and Vascular Endothelial Growth Factor (VEGF). Cancer Lett.

[33] Chen L., Tian G., Shao C., Cobos E., Gao W. (2010). Curcumin modulates eukaryotic initiation factors in human lung adenocarcinoma epithelial cells. Mol. Biol. Rep.

[34] Fu S., Kurzrock R. (2010). Development of curcumin as an epigenetic agent. Cancer.

[35] Lev-Ari S., Vexler A., Starr A., Ashkenazy-Voghera M., Greif J., Aderka D., Ben-Yosef R. (2007). Curcumin augments gemcitabine cytotoxic effect on pancreatic addenocarcinoma cell lines. Cancer Invest.

[36] Kunnumakkara A.B., Guha S., Krishnan S., Diagaradjane P., Gelovani J., Aggarwal B.B. (2007). Curcumin potentiates antitumor activity of gemcitabin in an orthotopic model of pancreatic cancer through suppression of proliferation, angiogenesis, and inhibition of nuclear factor-kappaB-regulated gene products. Cancer Res.

[37] Seol D.W., Chen Q., Zarnegar R. (2000). Transcriptional activation of the hepatocyte growth factor receptor (c-met) gene by its ligand (hepatocyte growth factor) is mediated through AP-1. Oncogene.

[38] Chen A., Xu J., Johnson A.C. (2006). Curcumin inhibits human colon cancer cell growth by suppressing gene expressions of epidermal growth factor receptor through reducing the activity of the transcription factor Egr-1. Oncogene.

[39] Chadalapaka G., Jutooru I., Burghardt R., Safe S. (2010). Drugs that target specificity proteins downregulate epidermal growth factor receptor in bladder cancer cells. Mol. Cancer Res.

[40] Lee J.Y., Lee Y.M., Chang G.C., Yu S.L., Hsieh W.Y., Chen J.J., Chen H.W., Yang P.C. (2011). Curcumin induces EGFR degradation in lung adenocarcinoma and modulates p38 activation in intestine: The versatile adjuvant for gefitinib therapy. PLoS One.

[41] Croce C.M. (2009). Causes and consequence of miRNA dysregulation in cancer. Nat. Rev. Genet.

[42] Valencia-Sanchez M.A., Liu J., Hannon G.J., Parker R. (2006). Control of translation and mRNA degradation by miRNAs and siRNAs. Genes Dev.

[43] Chen C.Z. (2005). MicroRNAs as oncogenes and tumor suppressors. N. Engl. J. Med.

[44] Ambros V. (2003). MicroRNA pathways in flies and worms: Growth, death, fat, stress, and timing. Cell.

[45] Bartel D.P. (2004). MicroRNAs: Genomics, biogenesis, mechanism, and function. Cell.

[46] Calin G.A., Dumitru C.D., Shimizu M., Bichi R., Zupo S., Noch E., Aldler H., Rattan S., Keating M., Rai K. (2002). Frequent deletions and down-regulation of micro-RNA genes miR15 and miR16 at 13q14 in chronic lymphocytic leukemia. Proc. Natl. Acad. Sci. USA.

[47] Esquela-Kerscher A., Slack F.J. (2006). Oncomirs—MicroRNAs with a role in cancer. Nat. Rev. Cancer.

[48] Calin G.A., Croce C.M. (2006). MicroRNA signature in human cancer. Nat. Rev. Cancer.

[49] Kumar M.S., Lu J., Mercer K.L., Golub T.R., Jacks T. (2007). Impaired microRNA processing enhances cellular transformation and tumorigenesis. Nat. Genet.

[50] Takamizawa J., Konishi H., Yanagisawa K., Tomida S., Osada H., Endoh H., Harano T., Yatabe Y., Nagino M., Nimura Y. (2004). Reduced expression of the *let-7* microRNAs in human lung cancers in association with shortened postoperative survival. Cancer Res.

[51] Hayashita Y., Osada H., Tatematsu Y., Yamada H., Yanagisawa K., Tomida S., Yatabe Y., Kawahara K., Sekido Y., Takahashi T. (2005). A polycistronic microRNA cluster, miR-17-92, is overexpressed in human lung cancers and enhances cell proliferation. Cancer Res.

[52] Osada H., Takahashi T. (2011). *let-7* and *miR-17-92*: Small-sized major players in lung cancer development. Cancer Sci.

[53] Yu L., Todd N.W., Xing L., Xie Y., Zhang H., Liu Z., Fang H., Zhang J., Katz R.L., Jiang F. (2010). Early detection of lung adenocarcinoma in sputum by a panel of microRNA markers. Int. J. Cancer.

[54] Foss K.M., Sima C., Ugolini D., Neri M., Allen K.E., Weiss G.J. (2011). miR-1254 and miR-574-5p: Serum-based microRNA biomarkers for early-stage non-small cell lung cancer. J. Thorac. Oncol.

[55] Yu S.L., Chen H.Y., Chang G.C., Chen C.Y., Chen H.W., Singh S., Cheng C.L., Yu C.J., Lee Y.C., Chen H.S. (2008). MicroRNA signature predicts survival and relapse in lung cancer. Cancer Cell.

[56] Patnaik S., Kannisto E., Knudsen S., Yendamuri S. (2010). Evaluation of microRNA expression profiles that may predict recurrence of localized stage I non-small cell lung cancer after surgical resection. Cancer Res.

[57] Matsubara H., Takeuchi T., Nishikawa E., Yanagisawa K., Hayashita Y., Ebi H., Yamada H., Suzuki M., Nagino M., Nimura Y. (2007). Apoptosis induction by antisense oligonucleotides against miR-17-5p and miR-20a in lung cancers overexpressing miR-17-92. Oncogene.

[58] Sun M., Estrov Z., Ji Y., Coombes K.R., Harris D.H., Kurzrock R. (2008). Curcumin (diferuloylmethane) alters the expression profiles of microRNAs in human pancreatic cancer cells. Mol. Cancer Ther.

[59] Zhang J., Du Y., Wu C., Ren X., Ti X., Shi J., Zhao F., Yin H. (2010). Curcumin promotes apoptosis in human adenocarcinoma cells through miR-186* signaling pathway. Oncol. Rep.

[60] Mudduluru G., George-William J.N., Muppala S., Asangani I.A., Kumarswamy R., Nelson L.D., Allgayer H. (2011). Curcumin regulates miR-21 expression and inhibits invasion and metastasis in colorectal cancer. Biosci. Rep.

[61] Yang J., Cao Y., Sun J., Zhang Y. (2010). Curcumin reduces the expression of Bcl-2 by upregulating miR-15a and miR-16 in MCF-7 cells. Med. Oncol.

[62] Guo L., Liu Y., Bai Y., Sun Y., Xiao F., Guo Y. (2010). Gene expression profiling of drug-resistant small cell lung cancer cells by combining microRNA and cDNA expression analysis. Eur. J. Cancer.

[63] Garofalo M., Quintavalle C., di Leva G., Zanca C., Romano G., Taccioli C., Liu C.G., Croce C.M., Condorelli G. (2008). MicroRNA signatures of TRAIL resistance in human non-small cell lung cancer. Oncogene.

[64] Zhu W., Shan X., Wang T., Shu Y., Liu P. (2010). MiR-181b modulates multidrug resistance by targeting BCL2 in human cancer cell lines. Int. J. Cancer.

[65] Zhang J., Zhang T., Ti X., Shi J., Wu C., Ren X., Yin H. (2010). Curcumin promotes apoptosis in A549/DDP multidrug-resistant human lung adenocarcinoma through an miRNA signaling pathway. Biochem. Biophys. Res. Commun.

[66] Ali S., Ahmad A., Banerjee S., Padhye S., Dominiak K., Schaffert J.M., Wang Z., Philip P.A., Sarkar F.H. (2010). Gemcitabine sensitivity can be induced in pancreatic cancer cells through modulation of miR-200 and miR-21 expression by curcumin or its analogue CDF. Cancer Res.

[67] Baehrecke E.H. (2002). How death shapes life during development. Nat. Rev. Mol. Cell Biol.

[68] Jaboin J.J., Hwang M., Lu B. (2009). Autophagy in lung cancer. Method Enzymol.

[69] Canuto R.A., Tessitore L., Muzio G., Autelli R., Baccino F.M. (1993). Tissue protein turnover during liver carcinogenesis. Carcinogenesis.

[70] Schwartz L.M., Smith S.W., Jones M.E., Osborne B.A. (1993). Do all programmed cell deaths occur via apoptosis?. Proc. Natl. Acad. Sci. USA.

[71] Schwarze P.E., Seglen P.O. (1985). Reduced autophagic activity, improved protein balance and enhanced *in vitro* survival of hepatocytes isolated from carcinogen-treated rats. Exp. Cell Res.

[72] Ryter S.W., Choi A.M.K. (2010). Autophagy in the lung. Proc. Am. Thorac. Soc.

[73] Levine B. (2006). Unraveling the role of autophagy in cancer. Autophagy.

[74] Kondo Y., Kondo S. (2006). Autophagy and cancer therapy. Autophagy.

[75] Gozuacik D., Kimchi A. (2007). Autophagy and cell death. Curr. Top. Dev. Biol.

[76] Kim K.W., Hwang M., Moretti L., Jaboin J.J., Cha Y.I., Lu B. (2008). Autophagy upregulation by inhibitors of caspase-3 and mTOR enhances radiotherapy in a mouse model of lung cancer. Autophagy.

[77] Ding X.L., Zhang H.Y., Qi L., Zhao B.X., Lian S., Lv H.S., Miao J.Y. (2009). Synthesis of novel pyrazole carboxamide derivatives and discovery of modulators for apoptosis or autophagy in A549 lung cancer cells. Bioorg. Med. Chem. Lett.

[78] Zheng L.W., Li Y., Ge D., Zhao B.X., Liu Y.R., Lv H.S., Ding J., Miao J.Y. (2010). Synthesis of novel oxime-containing pyrazole derivatives and discovery of regulators for apoptosis and autophagy in A549 lung cancer cells. Bioorg. Med. Chem. Lett.

[79] Han W., Pan H., Chen Y., Sun J., Wang Y., Li J., Ge W., Feng L., Lin X., Wang X. (2011). EGFR tyrosine kinase inhibitors activate autophagy as a cytoprotective response in human lung cancer cells. PLoS One.

[80] Viola G., Bortolozzi R., Hamel E., Moro S., Brun P., Castagliuolo I., Ferlin M.G., Basso G. (2012). MG-2477, a new tubulin inhibitor, induces autophagy through inhibition of the Akt/mTOR pathway and delayed apoptosis in A549 cells. Biochem. Pharmacol.

[81] He Q., Huang B., Zhao J., Zhang Y., Zhang S., Miao J. (2008). Knockdown of integrin β4-induced autophagic cell death associated with P53 in A549 lung adenocarcinoma cells. FEBS J.

[82] Jia Y.L., Li J., Qin Z.H., Liang Z.Q. (2009). Autophagic and apoptotic mechanisms of curcumin-induced death in K562 cells. J. Asian Nat. Prod. Res.

[83] O’Sullivan-Coyne G., O’Sullivan G.C., O’Donovan T.R., Piwocka K., McKenna S.L. (2009). Curcumin induces apoptosis-independent death in oesophageal cancer cells. Br. J. Cancer.

[84] Kabeya Y., Mizushima N., Ueno T., Yamamoto A., Kirisako T., Noda T., Kominami E., Ohsumi Y., Yoshimori T. (2000). LC3, a mammalian homologue of yeast Apg8p, is localized in autophagosome membranes after processing. EMBO J.

[85] Aoki H., Takada Y., Kondo S., Sawaya R., Aggarwal B.B., Kondo Y. (2007). Evidence that curcumin suppresses the growth of malignant gliomas *in vitro* and *in vivo* through induction of autophagy: Role of Akt and extracellular signal-regulated kinase signaling pathways. Mol. Pharmacol.

[86] Shinojima N., Yokoyama T., Kondo Y., Kondo S. (2007). Roles of the Akt/mTOR/p70S6K and ERK1/2 signaling pathways in curcumin-induced autophagy. Autophagy.

[87] Qian H., Yang Y., Wang X. (2011). Curcumin enhanced adriamycin-induced human liver-derived Hepatoma G2 cell death through activation of mitochondria-mediated apoptosis and autophagy. Eur. J. Pharm. Sci.

[88] Wilken R., Veena M.S., Wang M.B., Srivatsan E.S. (2011). Curcumin: A review of anti-cancer properties and therapeutic activity in head and neck squamous cell carcinoma. Mol. Cancer.

[89] Bonnet D., Dick J.E. (1997). Human acute myeloid leukemia is organized as a hierarchy that originates from a primitive hematopoietic cell. Nat. Med.

[90] Maitland N.J., Collins A.T. (2010). Cancer stem cells—A therapeutic target?. Curr. Opin. Mol. Ther.

[91] Hermann P.C., Bhaskar S., Cioffi M., Heeschen C. (2010). Cancer stem cells in solid tumors. Semin. Cancer Biol.

[92] Chen S.Y., Huang Y.C., Liu S.P., Tsai F.J., Shyu W.C., Lin S.Z. (2011). An overview of concepts for cancer stem cells. Cell Transplant.

[93] Clevers H. (2011). The cancer stem cell: Premises, promises and challenges. Nat. Med.

[94] Ho M.M., Ng A.V., Lam S., Hung J.Y. (2007). Side population in human lung cancer cell lines and tumors is enriched with stem-like cancer cells. Cancer Res.

[95] Levina V., Marrangoni A.M., DeMarco R., Gorelik E., Lokshin A.E. (2008). Drug-selected human lung cancer stem cells: Cytokine network, tumorigenic and metastatic properties. PLoS One.

[96] Kitamura H., Okudela K., Yazawa T., Sato H., Shimoyamada H. (2009). Cancer stem cell: Implications in cancer biology and therapy with special reference to lung cancer. Lung Cancer.

[97] Wu C., Alman B.A. (2008). Side population cells in human cancers. Cancer Lett.

[98] Sophos N.A., Vasiliou V (2003). Aldehyde dehydrogenase gene superfamily: The 2002 update. Chem. Biol. Interact.

[99] Zhang W.C., Shyh-Chang N., Yang H., Rai A., Umashankar S., Ma S., Soh B.S., Sun L.L., Tai B.C., Nga M.E. (2012). Glycine decarboxylase activity drives non-small cell lung cancer tumor-initiating cells and tumorigenesis. Cell.

[100] Teng Y., Wang X., Wang Y., Ma D. (2010). Wnt/beta-catenin signaling regulates cancer stem cells in lung cancer A549 cells. Biochem. Biophys. Res. Commun.

[101] Bertolini G., Roz L., Perego P., Tortoreto M., Fontanella E., Gatti L., Pratesi G., Fabbri A., Andriani F., Tinelli S. (2009). Highly tumorigenic lung cancer CD133^+^ cells display stem-like features and are spared by cisplatin treatment. Proc. Natl. Acad. Sci. USA.

[102] Riccioni R., Dupuis M.L., Bernabei M., Petrucci E., Pasquini L., Mariani G., Cianfriglia M., Testa U. (2010). The cancer stem cell selective inhibitor salinomycin is a p-glycoprotein inhibitor. Blood Cells Mol. Dis.

[103] Yilmaz O.H., Valdez R., Theisen B.K., Guo W., Ferguson D.O., Wu H., Morrison S.J. (2006). PTEN dependence distinguishes haematopoietic stem cells from leukaemia-initiating cells. Nature.

[104] Lin L., Fuchs J., Li C., Olson V., Bekaii-Saab T., Li J. (2011). STAT3 signaling pathway is necessary for cell survival and tumorsphere forming capacity in ALDH^+^/CD133^+^ stem cell-like human colon cancer cells. Biochem. Biophys. Res. Commun.

[105] Kakarala M., Brenner D.E., Korkaya H., Cheng C., Tazi K., Ginestier C., Liu S., Dontu G., Wicha M.S. (2010). Targeting breast stem cells with the cancer preventive compounds curcumin and piperine. Breast Cancer Res. Treat.

[106] Fong D., Yeh A., Naftalovich R., Choi T.H., Chan M.M. (2010). Curcumin inhibits the side population (SP) phenotype of the rat C6 glioma cell line: Towards targeting of cancer stem cells with phytochemicals. Cancer Lett.

[107] Lim K.J., Bisht S., Bar E.E., Maitra A., Eberhart C.G. (2011). A polymeric nanoparticle formulation of curcumin inhibits growth, clonogenicity and stem-like fraction in malignant brain tumors. Cancer Biol. Ther.

[108] Lin L., Liu Y., Li H., Li P.K., Fuchs J., Shibata H., Iwabuchi Y., Lin J. (2011). Targeting colon cancer stem cells using a new curcumin analogue, GO-Y030. Br. J. Cancer.

[109] Bao B., Ali S., Banerjee S., Wang Z., Logna F., Azmi A.S., Kong D., Ahmad A., Li Y., Padhye S. (2012). Curcumin analogue CDF inhibits pancreatic tumor growth by switching on suppressor microRNAs and attenuating EZH2 expression. Cancer Res.

[110] Jordan C.T. (2008). Can we finally target the leukemic stem cells?. Best Pract. Res. Clin. Haematol.

[111] Neelakantan S., Nasim S., Guzman M.L., Jordan C.T., Crooks P.A. (2009). Aminoparthenolides as novel anti-leukemic agents: Discovery of the NF-κB inhibitor, DMAPT (LC-1). Bioorg. Med. Chem. Lett.

[112] National Cancer Institute (1996). Clinical development plan: Curcumin. J. Cell Biochem. Suppl..

[113] Sharma R.A., Euden S.A., Platton S.L., Cooke D.N., Shafayat A., Hewitt H.R., Marczylo T.H., Morgan B., Hemingway D., Plummer S.M. (2004). Phase I clinical trial of oral curcumin: Biomarkers of systemic activity and compliance. Clin. Cancer Res.

[114] Cheng A.L., Hsu C.H., Lin J.K., Hsu M.M., Ho Y.F., Shen T.S., Ko J.Y., Lin J.T., Lin B.R., Ming-Shiang W. (2001). Phase I clinical trial of curcumin, a chemopreventive agent, in patients with high-risk or pre-malignant lesions. Anticancer Res.

[115] Wahlstrom B., Blennow G. (1978). A study on the fate of curcumin in the rat. Acta Pharmacol. Toxicol. (Copenh.).

[116] Shoba G., Joy D., Joseph T., Majeed M., Rajendran R., Srinivas P.S. (1998). Influence of piperine on the pharmacokinetics of curcumin in animals and human volunteers. Planta Med.

[117] Bisht S., Feldmann G., Soni S., Ravi R., Karikar C., Maitra A., Maitra A (2007). Polymeric nanoparticleencapsulated curcumin (“nanocurcumin”): A novel strategy for human cancer therapy. J. Nanobiotechnol.

[118] Li L., Braiteh F.S., Kurzrock R. (2005). Liposome-encapsulated curcumin: *In vitro* and *in vivo* effects on proliferation, apoptosis, signaling, and angiogenesis. Cancer.

[119] Maiti K., Mukherjee K., Gantait A., Saha B.P., Mukherjee P.K. (2007). Curcumin-phospholipid complex: Preparation, therapeutic evaluation and pharmacokinetic study in rats. Int. J. Pharm.

[120] Marczylo T.H., Verschoyle R.D., Cooke D.N., Morazzoni P., Steward W.P., Gescher A.J. (2007). Comparison of systemic availability of curcumin with that of curcumin formulated with phosphatidylcholine. Cancer Chemother. Pharmacol.

